# Exploring the potential of approved drugs for triple-negative breast cancer treatment by targeting casein kinase 2: Insights from computational studies

**DOI:** 10.1371/journal.pone.0289887

**Published:** 2023-08-14

**Authors:** Tagyedeen H. Shoaib, Walaa Ibraheem, Mohammed Abdelrahman, Wadah Osman, Asmaa E. Sherif, Ahmed Ashour, Sabrin R. M. Ibrahim, Kholoud F. Ghazawi, Samar F. Miski, Sara A. Almadani, Duaa Fahad ALsiyud, Gamal A. Mohamed, Abdulrahim A. Alzain

**Affiliations:** 1 Faculty of Pharmacy, Department of Pharmaceutical Chemistry, University of Gezira, Gezira, Sudan; 2 Faculty of Pharmacy, Department of Pharmaceutics, University of Gezira, Gezira, Sudan; 3 Faculty of Pharmacy, Department of Pharmacognosy, Prince Sattam Bin Abdulaziz University, Al-kharj, Saudi Arabia; 4 Faculty of Pharmacy, Department of Pharmacognosy, University of Khartoum, Khartoum, Sudan; 5 Faculty of Pharmacy, Department of Pharmacognosy, Mansoura University, Mansoura, Egypt; 6 Department of Chemistry, Preparatory Year Program, Batterjee Medical College, Jeddah, Saudi Arabia; 7 Faculty of Pharmacy, Department of Pharmacognosy, Assiut University, Assiut, Egypt; 8 Clinical Pharmacy Department, College of Pharmacy, Umm Al-Qura University, Makkah, Saudi Arabia; 9 Department of Pharmacology and Toxicology, College of Pharmacy, Taibah University, Al-Madinah Al-Munawwarah, Saudi Arabia; 10 Department of Medical Laboratories—Hematology, King Fahd Armed Forces Hospital, Corniche Road, Andalus, Jeddah, Saudi Arabia; 11 Department of Natural Products and Alternative Medicine, Faculty of Pharmacy, King Abdulaziz University, Jeddah, Saudi Arabia; kafrelsheikh University, EGYPT

## Abstract

Triple-negative breast cancer (TNBC) is an aggressive malignancy that requires effective targeted drug therapy. In this study, we employed *in silico* methods to evaluate the efficacy of seven approved drugs against human ck2 alpha kinase, a significant modulator of TNBC metastasis and invasiveness. Molecular docking revealed that the co-crystallized reference inhibitor 108600 achieved a docking score of (-7.390 kcal/mol). Notably, among the seven approved drugs tested, sunitinib, bazedoxifene, and etravirine exhibited superior docking scores compared to the reference inhibitor. Specifically, their respective docking scores were -10.401, -7.937, and -7.743 kcal/mol. Further analysis using MM/GBSA demonstrated that these three top-ranked drugs possessed better binding energies than the reference ligand. Subsequent molecular dynamics simulations identified etravirine, an FDA-approved antiviral drug, as the only repurposed drug that demonstrated a stable and reliable binding mode with the human ck2 alpha protein, based on various analysis measures including RMSD, RMSF, and radius of gyration. Principal component analysis indicated that etravirine exhibited comparable stability of motion as a complex with human ck2 alpha protein, similar to the co-crystallized inhibitor. Additionally, Density functional theory (DFT) calculations were performed on a complex of etravirine and a representative gold atom positioned at different sites relative to the heteroatoms of etravirine. The results of the DFT calculations revealed low-energy complexes that could potentially serve as guides for experimental trials involving gold nanocarriers of etravirine, enhancing its delivery to malignant cells and introducing a new drug delivery route. Based on the results obtained in this research study, etravirine shows promise as a potential antitumor agent targeting TNBC, warranting further investigation through experimental and clinical assessments.

## Introduction

In 2020, female breast cancer emerged as the most commonly diagnosed cancer, with an estimated 2.3 million new cases worldwide [1]. Notably, it also represents the leading cause of cancer incidence and mortality among women. Among the various types of breast cancer, triple-negative breast cancer (TNBC) accounts for approximately 10% to 15% of all diagnosed cases [2].

TNBC is characterized by the absence of estrogen receptor (ER), progesterone receptors (PR), and human epidermal growth factor receptors (HER2) expression [3–6]. Targeted therapeutic strategies have been successfully employed for the treatment of ER-positive and HER2-positive subtypes of breast cancer [7]. However, TNBCs do not respond to targeted therapies and are typically treated with nonselective chemotherapy drugs. TNBCs exhibit more aggressive clinical manifestations, higher rates of relapse, and the molecular mechanisms underlying relapse are not yet fully understood [8,9]. Consequently, TNBCs represent the most malignant form of breast cancer, necessitating the urgent discovery of novel targeted therapies [10,11].

Chemotherapy resistance in TNBC is a significant factor that negatively impacts patients’ prognosis and overall survival rates [12,13]. One of the main contributors to resistance in triple-negative breast cancer cells is the presence of breast cancer stem cells (BCSCs) within the tumour [14,15]. BCSCs possess unique properties that enable them to self-renew and promote tumour cell growth. Furthermore, the increased invasiveness and metastatic potential of TNBC are influenced by the molecular pathways involving kinases present in BCSCs [16,17].

Among the kinases present in BCSCs, human casein kinase (CK2) plays a crucial role. CK2 is a serine/threonine protein kinase that is abundantly expressed and involved in various cellular functions, including cell growth, proliferation, and differentiation. The CK2 holoenzyme consists of two subunits, alpha and beta, with CK2 alpha being one of them [18]. CK2 has been demonstrated to phosphorylate and inactivate the tumour suppressor protein p53, leading to uncontrolled cell growth and cancer initiation [19].

Cancer therapy is a rapidly evolving field, and one promising approach for the discovery of new anticancer agents is drug repurposing, which involves identifying new indications for existing and approved medications [20]. Recently, a study proposed a set of approved drugs as new leads against breast cancer using a computational neural graph model [21]. Accordingly, in our present study, we selected these approved drugs as candidates to be repurposed against TNBC.

In the field of *in silico* drug design, molecular modelling serves as a valuable approach for structure-based drug design. It relies on the three-dimensional structures of proteins and encompasses various methodologies, including molecular docking, molecular dynamics simulations, structure-based pharmacophore modelling, and quantum mechanics calculations [22].

Effective delivery of chemotherapeutic agents to target tumour sites remains a challenging task. Nanoparticle drug delivery systems (DDSs), such as gold nanoparticles, have emerged as promising strategies to enhance drug accumulation in tumours while minimizing adverse effects [23]. Gold nanoparticles, in particular, serve as useful transport vehicles due to their ability to improve overall clinical outcomes and reduce side effects associated with chemotherapy [24,25].

The objective of this research was to repurpose already approved drugs for the treatment of TNBC using *in silico* drug discovery approaches. The selection of the candidate drugs was based on careful scrutiny of the safety profile and the convenience of the original indication. Additionally, the study aimed to explore the potential of employing gold nanoparticles to deliver a set of approved drugs against TNBC. A theoretical analysis was conducted to identify the most favourable interaction site with a gold surface, which would facilitate the availability of the drug for binding to the active site. DFT calculations were employed as a virtual microscope to understand and elucidate the nature of interactions between drugs and the supercell of the gold surface in the nanoparticle delivery system [26–32]. Furthermore, the utilization of DFT calculations with a single gold atom served as a starting point for future experiments involving larger gold clusters as representatives of nanocarriers.

## Materials and methods

### Computational resources

The *in silico* studies were performed using Maestro v12.8, a molecular modelling software developed by Schrödinger Inc. Maestro offers a range of tools for various molecular modelling tasks, including protein structure prediction, ligand docking, molecular dynamics simulations, and analysis of simulation results. For molecular dynamics (MD) simulations, GROMACS 2022.2 was employed. GROMACS is an open-source simulation software widely utilized in computational chemistry for studying biomolecules through MD simulations. It is known for its efficiency, high performance, and user-friendly interface. DFT calculations were conducted using Gaussian 16 software. Gaussian 16 is a popular software extensively used in computational chemistry for performing diverse electronic structure calculations, including DFT. It provides a comprehensive set of tools for the interpretation, evaluation, and visualization of the results obtained from these calculations.

### Protein preparation

The protein structure of human ck2 alpha kinase with a PDB ID (7L1X) was obtained from the RCSB Protein Data Bank [33] for docking simulations. The protein was prepared for the docking process using the Protein Preparation Wizard in the Schrödinger suite [34]. This tool facilitated the addition of missing hydrogen atoms, construction of missing residues and loops, resolution of atom overlaps, assignment of missing bond orders, determination of ligand protonation states, and optimization of the hydrogen-bonding network. Water molecules present in the crystal structure were removed, and the orientations of water molecules within a 3-angstrom range of the co-crystallized ligand were optimized and retained [35]. Subsequently, a receptor grid was generated using the coordinates of the co-crystallized ligand as the binding pocket for the docking procedure.

### Ligands preparation

**Fig 1:**
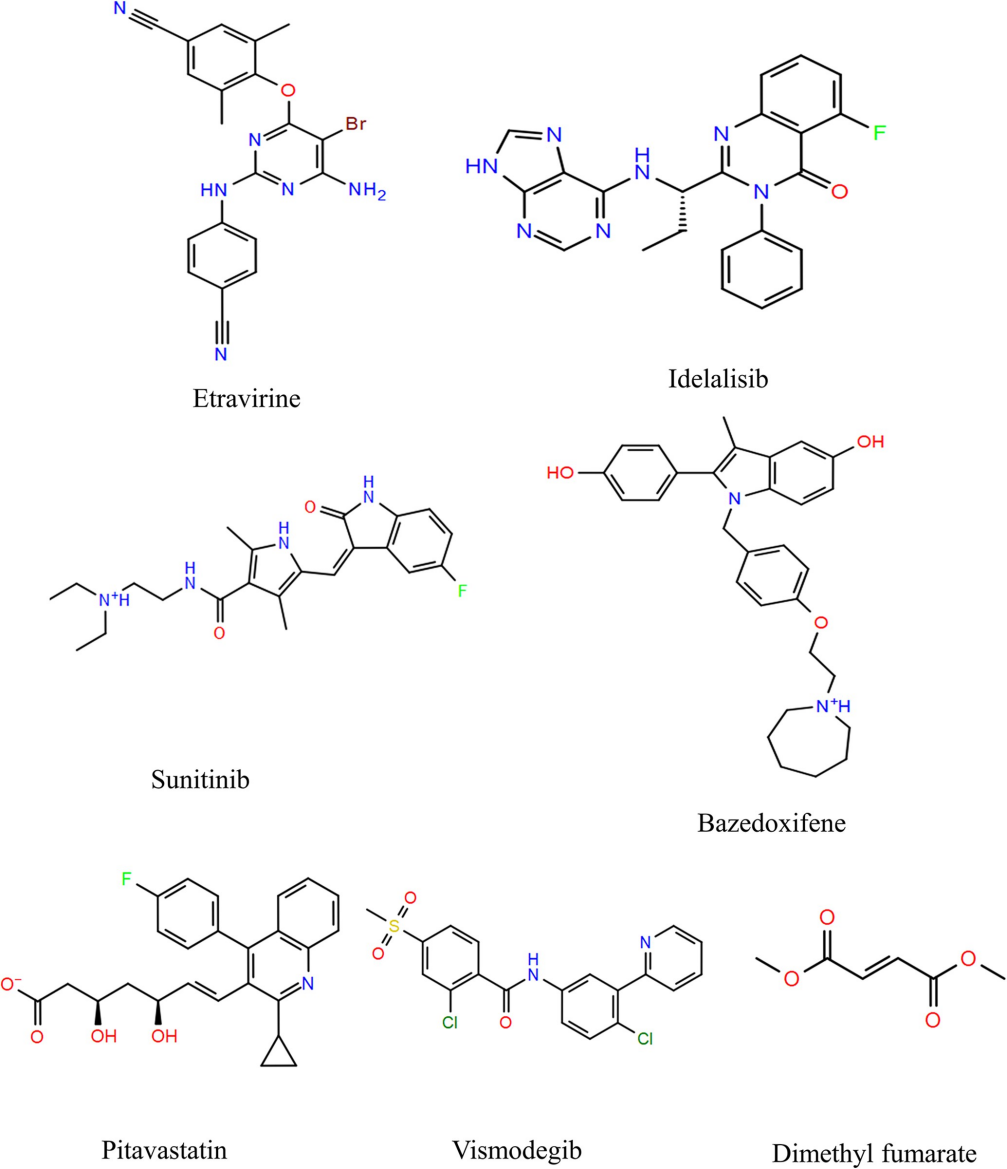
Chemical structures of the approved drugs.

The 3D structures of the approved drugs listed in Table 1 were obtained from PubChem [33] and prepared using the LigPrep tool. The chemical structures of these approved drugs are depicted in Fig 1. LigPrep is a software tool that generates accurate, energy-minimized 3D molecular structures. It incorporates advanced rules to rectify Lewis structures and eliminate errors in ligands, thereby reducing computational inaccuracies. Additionally, LigPrep offers the option to expand tautomeric and ionization states, ring conformations, and stereoisomers, allowing for the generation of diverse chemical and structural variations from a single input structure [36].

**Table 1:** List of approved drugs repurposed against TNBC.

	Drug name	Origin indication
**1**	Etravirine	Human Immunodeficiency Virus type 1 infection.
**2**	Pitavastatin	Primary Hyperlipidemia
**3**	Sunitinib	Advanced Renal Cell Carcinoma
**4**	Idelalisib	Chronic Lymphocytic Leukemia; Relapsed Follicular B-cell non-Hodgkin Lymphoma; Relapsed Small Lymphocytic Lymphoma
**5**	Dimethyl fumarate	Multiple Sclerosis
**6**	Bazedoxifene	prevention of osteoporosis
**7**	Vismodegib	Locally Advanced or Metastatic Basal Cell Carcinoma

### Molecular docking and MM/GBSA binding free energy calculations

After the preparation of both ligands and protein, molecular docking was performed using the Glide module [37,38]. Glide offers various docking modes, including HTVS (high-throughput virtual screening), SP (standard precision), and XP (extra precision), each providing a different balance of accuracy and efficiency. A scoring function was employed to assess the strength of the binding affinity in the docking process [39–42].

Next, the Prime module was utilized to calculate the MM/GBSA (molecular mechanics/generalized Born surface area) values for the top docking poses [43,44]. The Prime module within the Schrödinger suite provides tools for conducting MM/GBSA calculations, which estimate the binding energies of ligand-receptor complexes. This process involves preparing input files, setting up the calculation in Maestro, executing the calculation using the Prime MM/GBSA tool, and analysing the output to obtain binding free energy estimates and identify significant energetic contributions.

### Molecular dynamics (MD) simulation

The most favourable docking configurations of the ck2 alpha protein complexed with the repurposed drugs and the co-crystallized reference ligand were selected for the subsequent MD simulations. The MD simulations were conducted using the GROMACS 2022.2 software package [45]. The ligand topologies were generated using the LigParGen server [46–48], while the protein parameters were generated using the OPLS-aa force field and SPC/E water model within GROMACS.

A cubic simulation box was created around the protein-ligand complexes using the gmx editconf module. The box was set to a cubic shape, and the protein-drug complex was positioned at the center with a distance of 1.0 nanometres from the box’s edge. In the case of etravirine, the simulation box required a single Na ion and 22,947 water molecules to fully solvate the system. Bazedoxifene, being a neutral system, required 22,951 water molecules without the need for additional ions. In the case of inhibitor 108600, 22,945 water molecules and two Na ions were added to fill the simulation box.

Following box preparation, the system underwent energy minimization using the steepest descent algorithm. Subsequently, NVT (constant number of particles, volume, and temperature) and NPT (constant number of particles, pressure, and temperature) equilibration runs were performed for 100 ns to achieve a stable system at the desired temperature and pressure. The stability of the complexes was assessed using root mean square deviation (RMSD), root mean square fluctuation (RMSF), radius of gyration (Rg), and principal component analysis (PCA) along the simulation trajectories.

To analyze the MD trajectories, the built-in modules of GROMACS were utilized. The gmx rms command was used to calculate RMSD, gmx rmsf for calculating RMSF, and gmx gyrate for calculating the radius of gyration. The Graphing, Advanced Computation, and Exploration software (GRACE) was employed for generating the graphs. RMSD was calculated as a function of time, providing information about the deviation between the current coordinates and the reference coordinates at each time step. RMSF was calculated specifically for the C-alpha atoms, providing insights into the flexibility of the protein backbone residues. The radius of gyration was calculated at each time point, reflecting changes in the size and shape of the molecule throughout the simulation.

### DFT calculations

DFT calculations for etravirine were performed using Gaussian 16 software with GaussView 6 as the graphical interface. Etravirine was selected for these calculations based on its favourable docking score and stable MD profiles [49,50]. The 3D structure of etravirine was imported, and optimization and frequency parameters were applied using the B3LYP functional and the LanL2DZ basis set. In addition to etravirine, a gold atom was included in the calculations. Its 3D structure was drawn, and optimization and frequency parameters were generated using the B3LYP functional and the LanL2DZ basis set. The interaction between the gold atom and the surface heteroatoms of etravirine was subsequently examined.

## Results and discussion

### Molecular docking and MM/GBSA calculations

The docking scores and binding interactions of the selected drugs with human ck2 alpha kinase were analysed and compared to the co-crystallized ligand. The co-crystallized reference ligand, known as inhibitor 108600, is a novel multi-kinase inhibitor designed to target TNBC growth. Experimental studies have shown that this inhibitor reduces the viability of TNBC stem cells, induces unfavourable conformational changes in the human ck2 alpha enzyme, triggers apoptosis of TNBC stem cells, inhibits the growth of chemotherapy-resistant stem cells, and demonstrates efficacy in inhibiting TNBC growth both as a standalone treatment and in combination with other chemotherapeutic agents. Importantly, it synergizes with paclitaxel, thereby inhibiting metastatic TNBC *in vivo* [51].

The docking results indicated that sunitinib, bazedoxifene, and etravirine exhibited docking scores of -10.401, -7.937, and -7.743 kcal/mol, respectively. The co-crystallized reference ligand displayed a slightly lower docking score of -7.390 kcal/mol, as presented in Table 2.

**Table 2:** The docking scores and Molecular interactions of the docked approved drugs with ck2 alpha and their MM/GBSA.

Ligand	Hydrogen Bonds	Hydrophobic Interactions*		Binding Energy (Kcal/mol)	MM/ GBSA (Kcal/mol)
	Residue	Distance (Å)			
**Sunitinib**	VAL-116 VAL-116 GLU-114	1.99 Å 2.44 Å 2.65 Å	LEU-45, VAL-53, VAL66, ILE-95, PHE-113, MET-163, ILE-174.	-10.401	-62.16
**Bazedoxifene**	VAL-116 LEU-45	1.91 Å 2.14 Å	LEU-45, VAL-53, VAL66, ILE-95, PHE-113, MET-163, ILE-174.	-7.937	-33.56
**Etravirine**	VAL-116	1.93 Å	LEU-45, VAL-53, VAL66, ILE-95, MET-163, ILE-174, PHE-113.	-7.743	-48.38
**Inhibitor 108600 (reference)**	Water bridge with GLU-81 and TRP-176	1.93 Å	LEU-45, VAL-53, VAL-66, ILE-95, PHE-113, MET-163, ILE-174	-7.390	-29.97
**Idelalisib**	VAL-116 Water 1 Water 2	2.15 Å 1.91 Å 2.05 Å	LEU-45, VAL-53, VAL-66, ILE-95, PHE-113, MET-163, ILE-174	-6.931	-33.33
**Pitavastatin**	SER-51 TYR-50 LYS-49 ASP-175	2.35 Å 2.00 Å 2.73 Å 2.34 Å	LEU-45, TYR-50, VAL-66, PHE-113, VAL-116, MET-163, ILE-174	-6.274	-34.67
**Vismodegib**	-	-	LEU-45, VAL-53, VAL-66, ILE-95, PHE-113, VAL-116, MET-163, ILE-174	-5.638	-39.19
**Dimethyl Fumarate**	Water bridge with GLU-81 and TRP-176	2.11 Å 1.80 Å	VAL-53, VAL-66, ILE-95, PHE-113, VAL-116, MET-163, ILE-174	-1.340	-34.83

* The distance of all the hydrophobic interactions of the residues was set to maximally 4.0 angstroms to all complexes.

**Fig 2:**
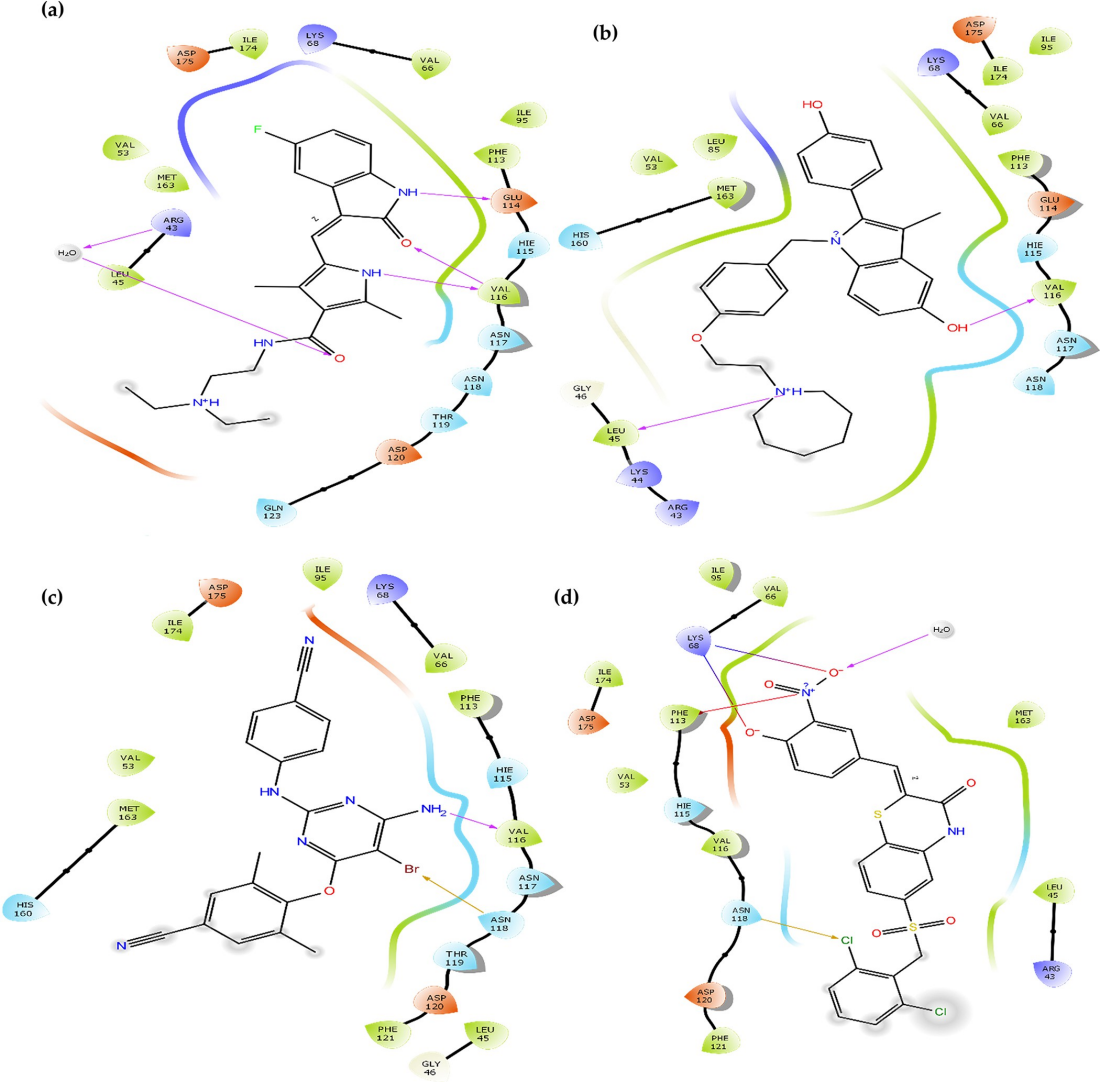
The two-dimensional (2D) interactions of the docked structures on the active pocket of human ck2 alpha catalytic subunit (PDB 7L1X): **(a)** Sunitinib **(b)** Bazedoxifene **(c)** Etravirine and **(d)** The co-crystallized reference inhibitor.

Upon analysing the binding interactions, it was observed that the top three drugs exhibited a common hydrogen bond with the amino acid residue VAL-116, indicating its significance in stabilizing the interaction within the binding pocket. In contrast, the reference ligand 108600 was stabilized by a water bridge that has simultaneously formed between the ligand and other two amino acid residues namely GLU-81 and TRP-176 (at a distance of 1.93 Å), and a salt bridge with amino acids PHE-113 and LYS-68. Additionally, it formed a halogen bond with ASN-118 that anchored the ligand in the binding pocket. Halogen bonds, which form between a halogen atom and other molecular entities like residual amino acids, can vary in strength, as presented in Fig 2. Notably, bromine bonds tend to be stronger than chlorine bonds [52]. In the case of etravirine compared to inhibitor 108600, the former established a bromine halogen bond with residue ASN-118, while the latter formed a chlorine halogen bond with the same residue. This indicates that etravirine binds more strongly to the active site compared to the potent inhibitor 108600 [51].

The binding mode of inhibitors to the active site of human ck2 alpha kinase depends on their orientations and the types of bonds they form. It is noteworthy that the top three approved drugs (sunitinib, bazedoxifene, and etravirine) and the inhibitor 108600 share similar binding modes and interactions with human ck2 protein. These results align well with previously studied inhibitors reported in the literature, particularly regarding hydrophobic interactions and hydrogen bonds with residues LEU-45, VAL-116, and ASN-118 [53,54].

To predict the end-point binding energy, molecular mechanics/generalized Born surface area (MM/GBSA) calculations were performed on the top docking poses and the reference ligand. MM/GBSA serves as a filter to reduce computational costs before conducting MD simulations [55]. As shown in Table 2, the MM/GBSA scores of the candidate compounds were significantly better than that of the reference ligand 108600, indicating a more favourable and stable binding affinity of the protein-ligand complex. In this context, a more negative score represents a stronger binding affinity [56]. MM/GBSA is a reliable tool for accurately determining ligand binding energies, surpassing the capabilities of molecular docking [57]. As a scoring function, MM/GBSA yields superior results [58]. However, it is recommended to follow MM/GBSA with MD simulations to gain a deeper understanding of the precise binding conformation of the ligands within the active site [57].

### Molecular dynamics simulation analysis

MD simulation trajectories provide valuable insights into the behaviour of proteins in the presence of small molecules [59]. In this study, MD simulations of the ck2 alpha protein were carried out for 100 ns, considering the co-crystallized ligand as a reference and the best poses of the docked approved drugs. The RMSD is a commonly used measure to assess the stability of a protein-ligand complex throughout the simulation time [60]. By employing the gmx rms module in GROMACS, the RMSD values of the complexes (i.e., protein backbone with etravirine, bazedoxifene, sunitinib and the co-crystallized reference ligand) were calculated and plotted on a graph, with simulation time on the x-axis and RMSD values on the y-axis, ranging from time zero to 100 ns. The RMSD values provide insights into the complex’s stability, where lower values indicate higher stability and fewer deviations from the reference mean distance [61].

**Fig 3:**
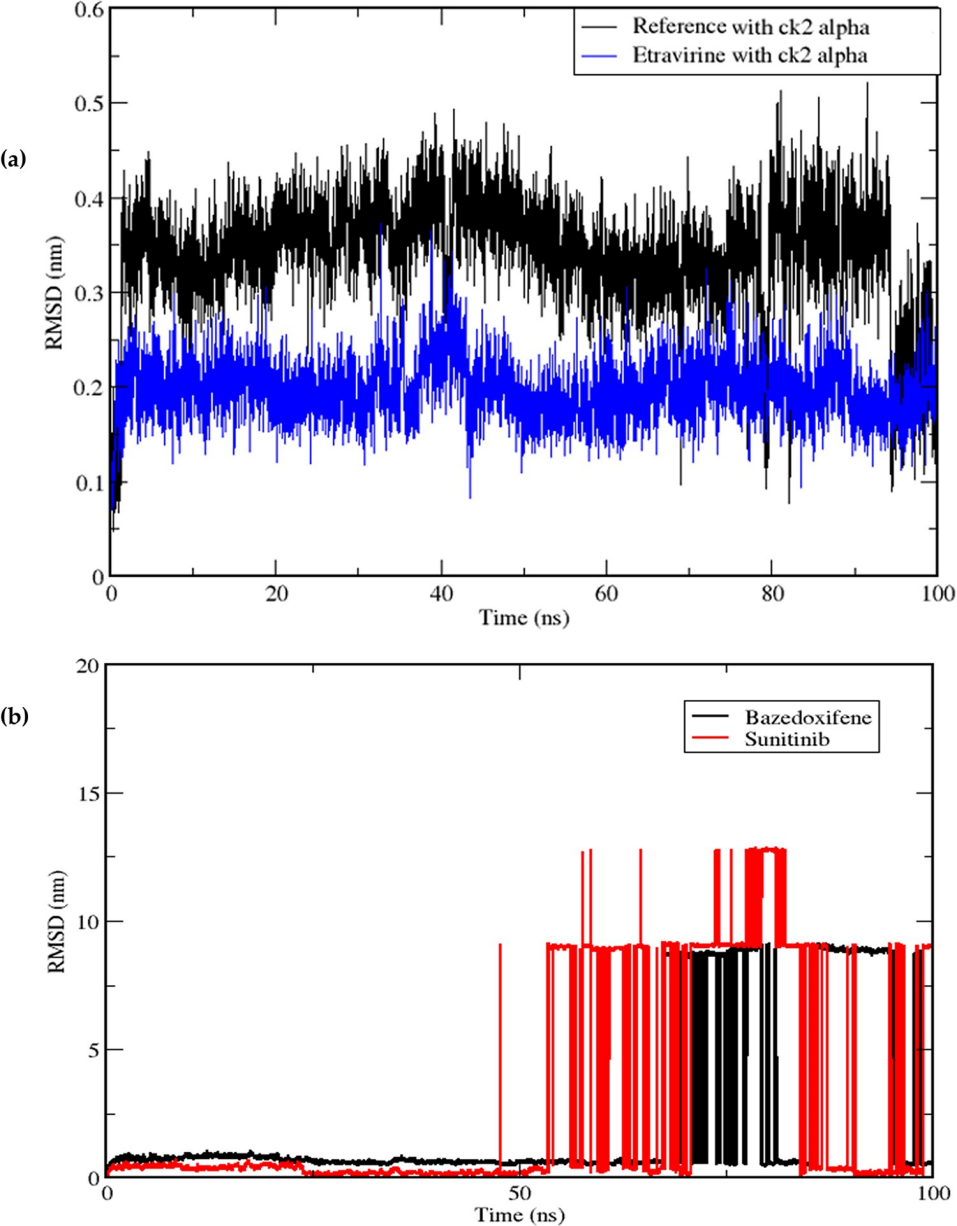
Displays: (a) Root mean square deviation (RMSD) graphical record of etravirine (Blue) and the reference ligand (Black) over a 100 ns simulation time, (b) Non-stable RMSD graphs of sunitinib (Red) and bazedoxifene (Black).

The RMSD graph in Fig 3(A) (Black) for the reference ligand demonstrated a relatively stable pattern with minimal fluctuations, ranging between 0.049 nm and 0.521 nm, and an average RMSD of 0.34 nm. On the other hand, among the three poses of the docked drugs, only etravirine in Fig 3(A) (Blue) exhibited a stable RMSD graph, indicating a stable protein-ligand complex. Etravirine displayed RMSD values of 0.071 nm and 0.373 nm, with an average of 0.197 nm, which is lower than that of the reference ligand. Further examination of the MD trajectories for stability using the Visual Molecular Dynamics (VMD) tool confirmed that both etravirine and the reference ligand remained within the simulation box throughout the simulation time. In contrast, the RMSD plot of sunitinib and bazedoxifene in Fig 3(B) showed non-stable RMSD graphs. Specifically, sunitinib converged at an RMSD below 0.5 nm but started to peak around the 57th ns, reaching a maximum RMSD value of 12.80 nm at around the 80th ns. The fluctuations observed in the graph indicate that the complexes are not stable for about half of the simulation time and tend to move out of the binding pocket and away from the simulation box, as observed in the trajectories visualized using VMD software.

To assess the stability of amino acid residues, RMSF analysis was performed on the protein complexed with the reference ligand and etravirine. RMSF is a statistical tool that quantifies the magnitude of residue motion during a simulation, providing insights into regions of the protein that exhibit significant fluctuations. The RMSF values of the protein main chain were plotted on the y-axis against the number of protein residues on the x-axis, using data generated by the gmx rmsf module in GROMACS. RMSF provides an estimation of the average deviation of the position of the residual amino acids from an energy-minimized reference structure.

**Fig 4:**
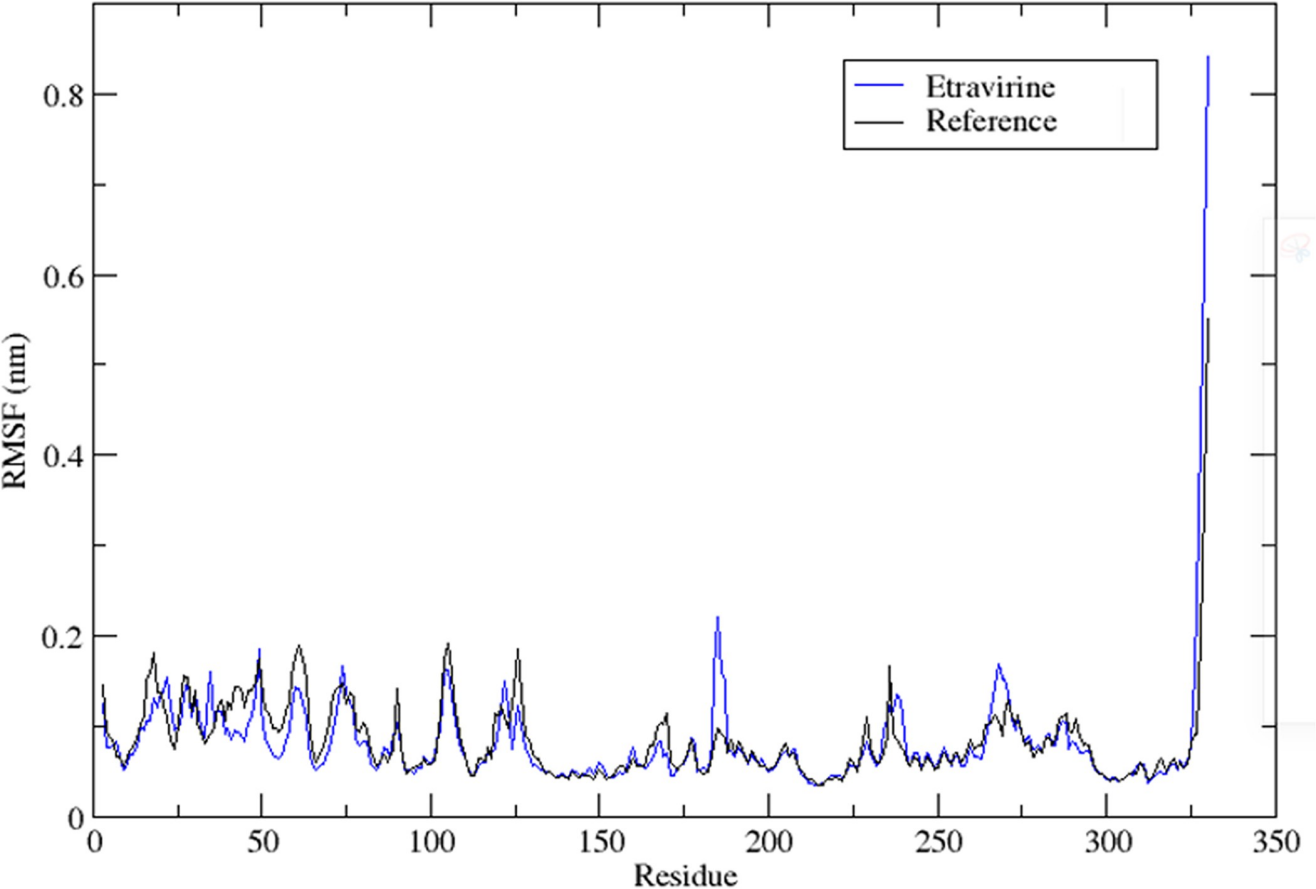
The root mean square fluctuation RMSF profile of etravirine (Blue) and the co-crystallized inhibitor (Black).

Fig 4 illustrates that the protein bound to the reference ligand and etravirine exhibited fewer fluctuations, with RMSF values below 0.2 nm. Notably, the regions with high RMSF values were distant from the binding pocket. This suggests that the active site can accommodate the bound drug without negatively affecting the stability of the binding.

**Fig 5:**
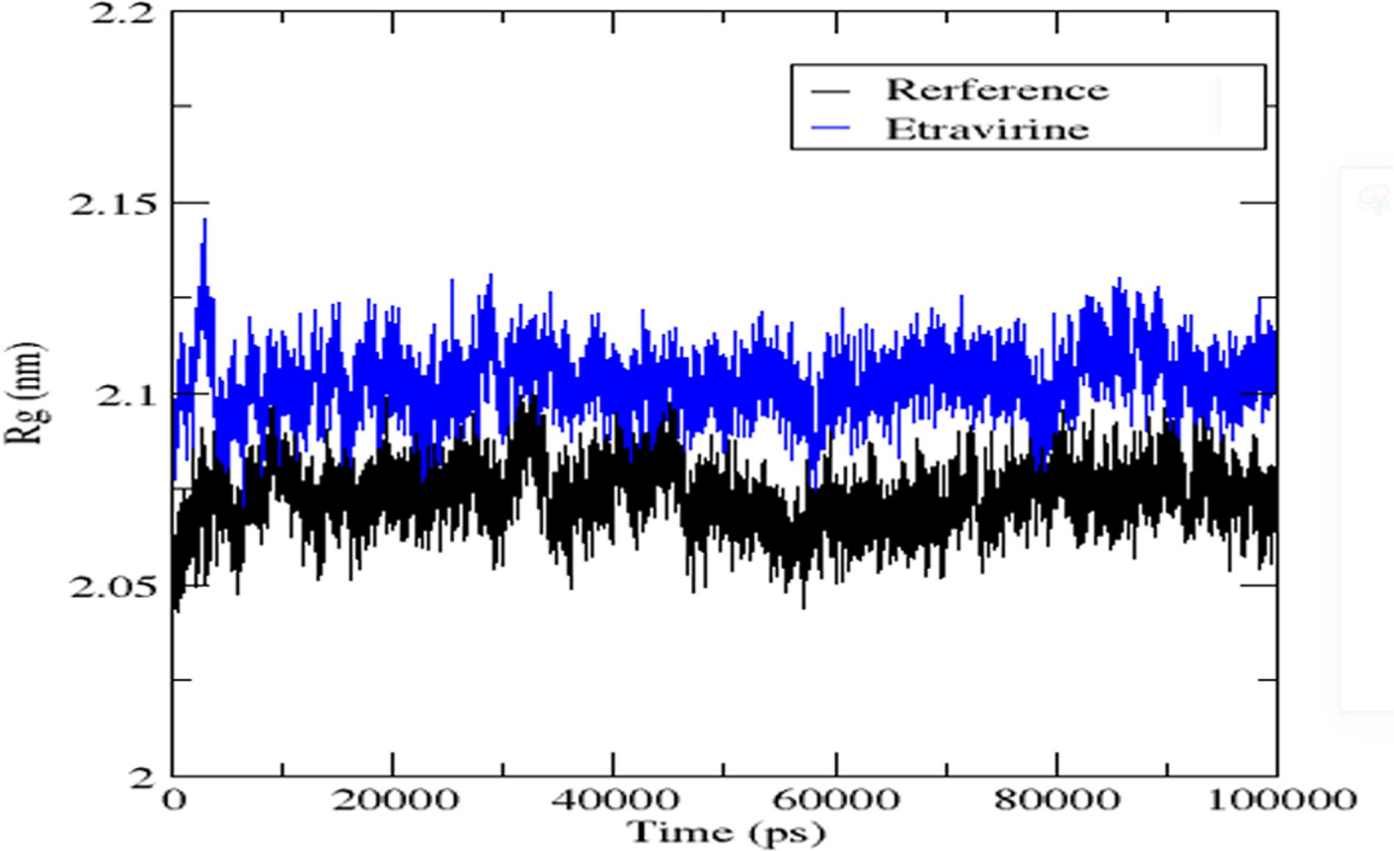
Radius of gyration graph of Etravirine (Blue) alongside the reference ligand (Black).

The radius of gyration (Rg) was employed as another parameter to confirm the findings of RMSD and RMSF regarding the stability of the protein-ligand complexes. Rg measures the distance between the centre of mass and the rotational axis of the ligand-bound protein assembly. It serves as an indicator of the compactness and stability of the protein structure during the simulation. The gmx gyrate option was utilized to generate data for the radius of gyration plot, whereby, the simulation time is plotted on the x-axis and the radius of gyration on the y-axis. Lower values of the radius of gyration indicate higher stability and compactness of the complex, while higher values suggest lower stability and compactness [62]. Analysing the radius of gyration values presented in Fig 5 for the protein bound to the reference ligand and etravirine, it is evident that the complexes exhibited small radius of gyration values, indicating higher stability. This finding is consistent with the results obtained from RMSD and RMSF analyses.

PCA was conducted on etravirine and compared to the reference ligand to further substantiate the stability of the complexes. PCA is commonly used to assess different modes of particle movement in MD trajectories [63,64]. For etravirine, which exhibited a stable MD profile, PCA analysis was performed to validate its stability through two-dimensional (2D) projection plots. For the reference inhibitor, a 2D projection plot was generated as well. The gmx covar code was used to calculate the covariance matrix of the alpha carbon atoms that were fitted to the complexed ligands. The matrix was then diagonalized to obtain the eigenvectors and eigenvalues. The gmx anaeig command was employed to analyse the eigenvectors (principal components, PCs), and PC1 and PC3 were selected to generate the 2D projection of the simulation’s trajectory.

**Fig 6:**
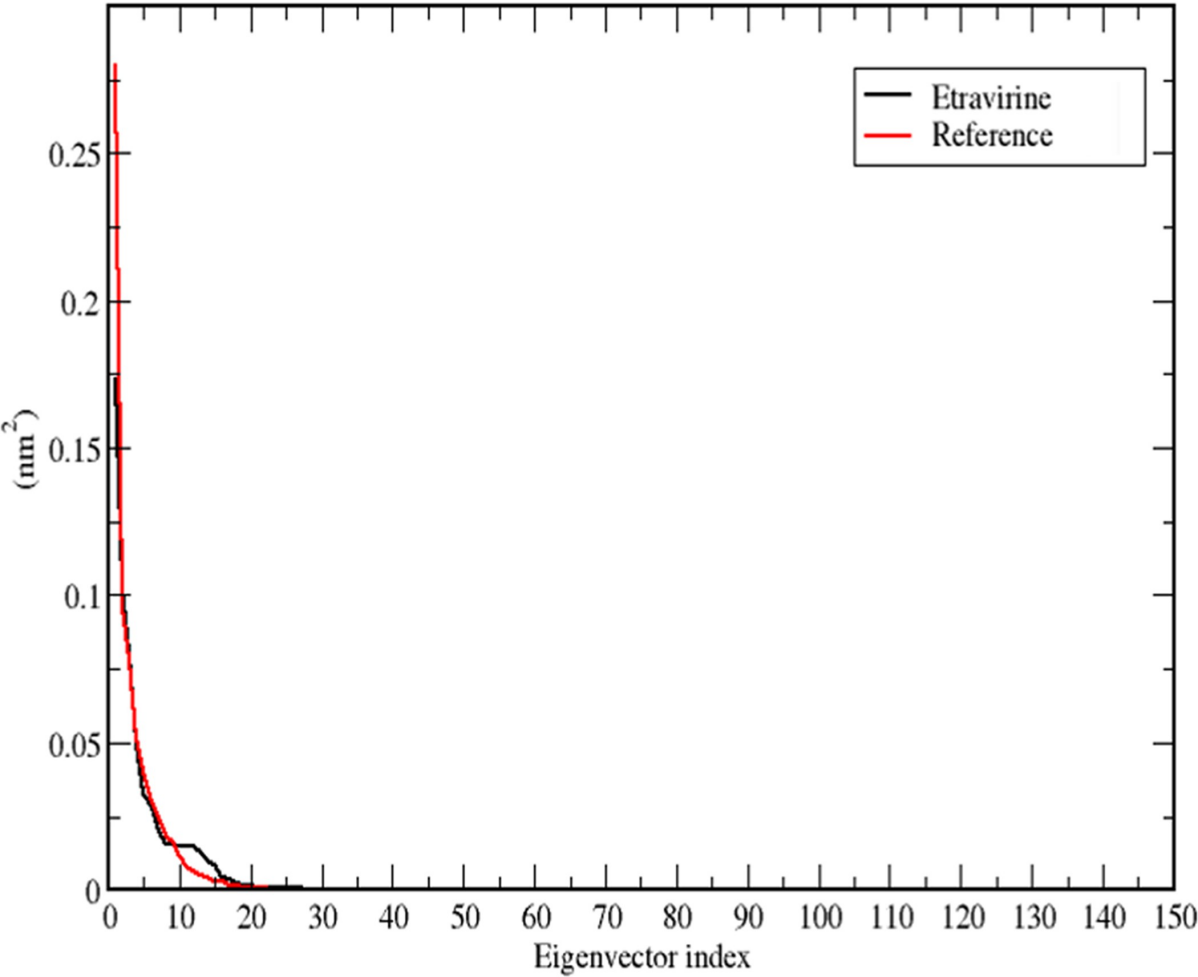
A plot that depicts the eigenvalues against eigenvectors for the drug etravirine (Black) and the reference ligand (Red).

For both etravirine and the reference ligand, it was observed that the first 10 eigenvectors accounted for more than 85% of the variance in the data, as depicted in Fig 6. This decreasing trend of variance against the related eigenvectors was obtained by diagonalizing the matrix of fluctuation covariance of the atoms. The eigenvalues for etravirine and the reference ligand were 0.62 nm^2^ and 0.69 nm^2^, respectively.

**Fig 7:**
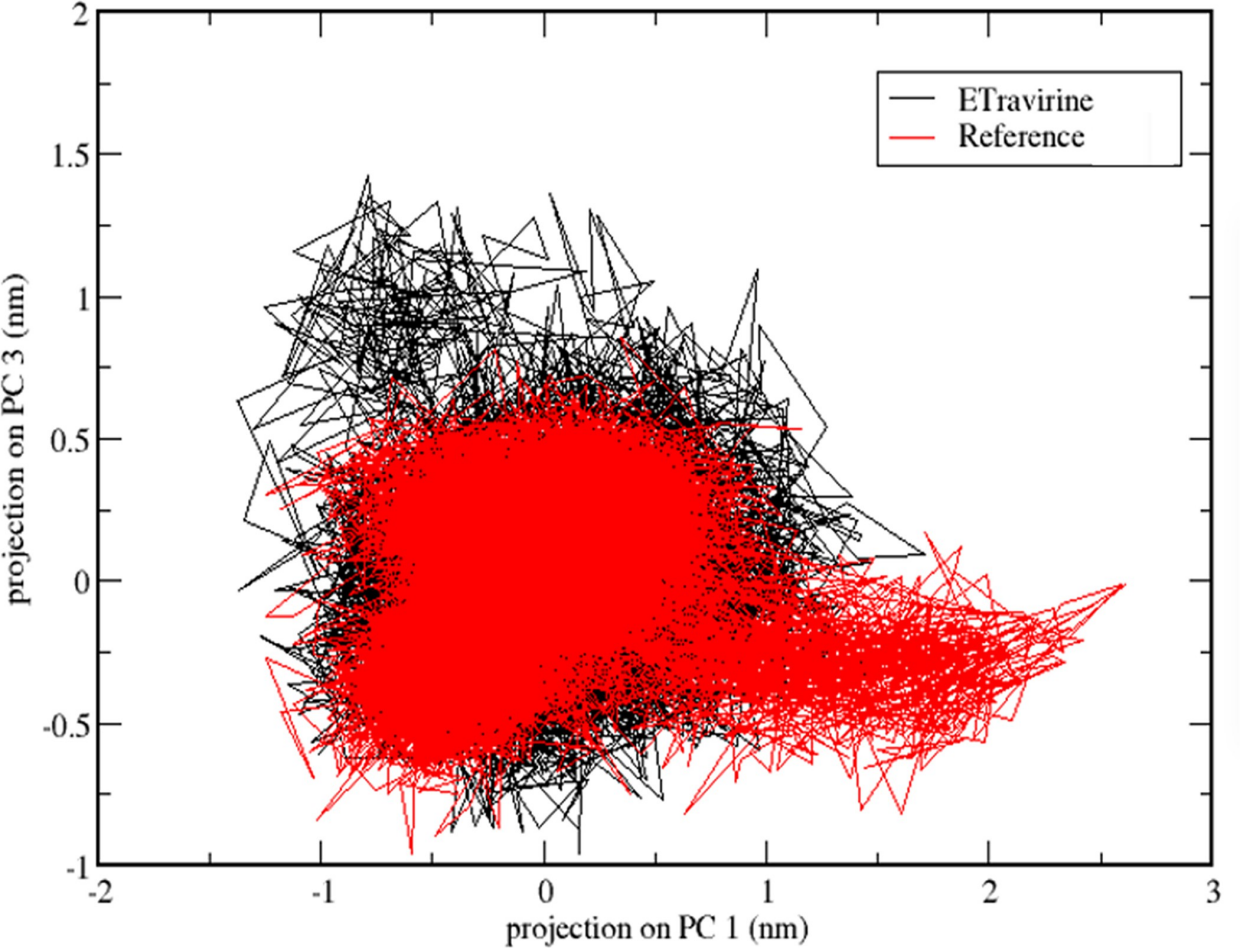
2D projection plot of the first and third principal components that are describing the dynamics of the protein-etravirine complex (Black) and protein-reference ligand complex (Red).

The dynamics of the complexes were further analysed using a two-dimensional projection plot based on the principal components PC1 and PC3. Fig 7 illustrates the projection of PC1 and PC3 for the reference molecule (in red) and etravirine (in black). In the analysis of the 2D projection plot, a stable complex is characterized by occupying a small area of the phase, whereas non-stable complexes tend to occupy a larger area [65]. Considering the candidate molecules, it is evident that they occupied a similar space, suggesting that they both present a comparable stability.

### DFT calculations

**Fig 8:**
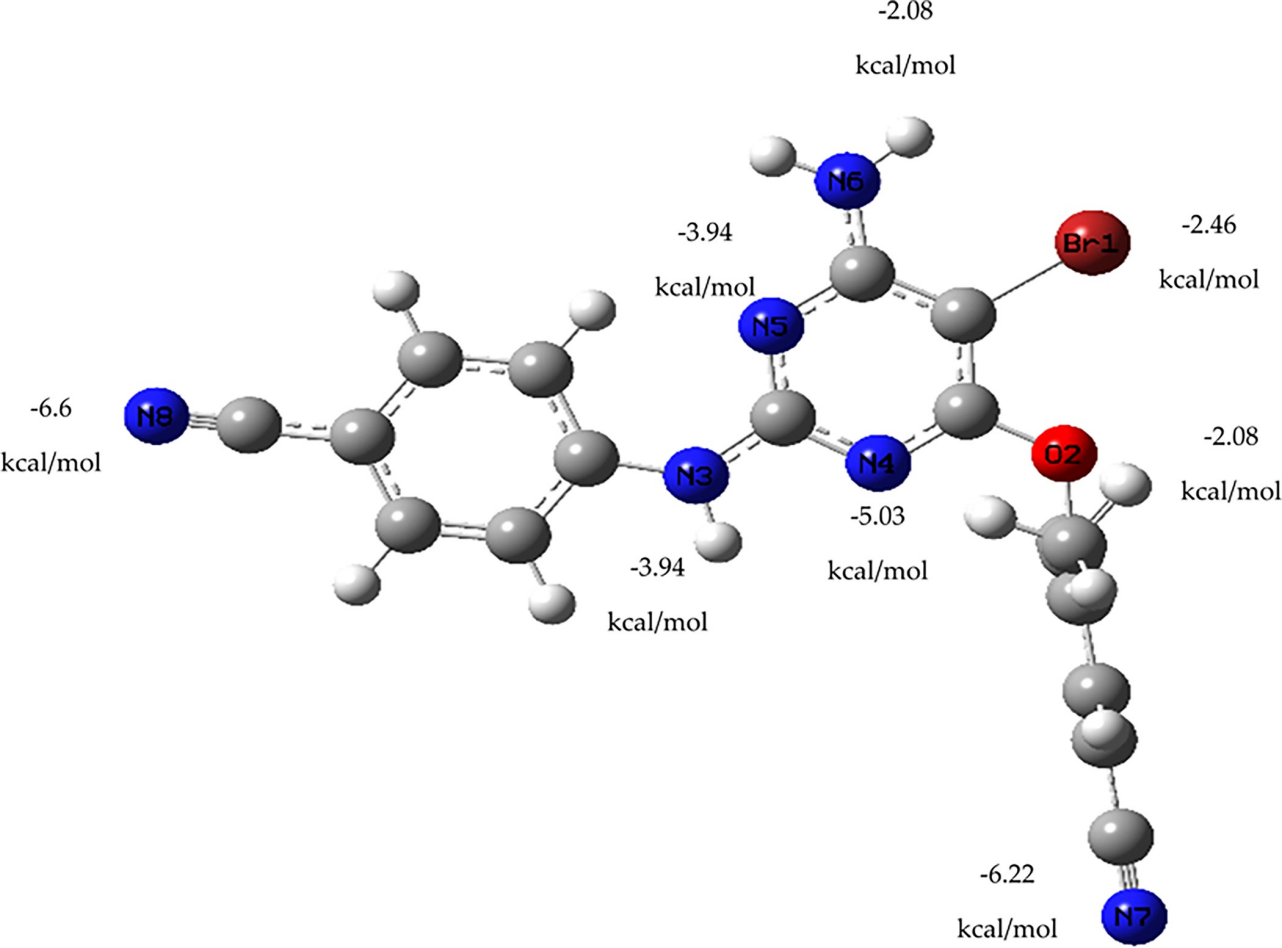
Summary of the different interaction energy values of a gold atom with the heteroatoms of etravirine.

The drug etravirine, which has been approved for use by the FDA, was subjected to DFT calculations after demonstrating a relatively better docking score than the co-crystallized ligand, along with a stable and reliable MD profile. The purpose of this quantum mechanical study was to evaluate the interaction energy between etravirine and a gold atom, which could assist in the design of gold nanoparticle carriers for this drug in the treatment of TNBC. Hence, Frequency and optimization were initially conducted on a single gold atom as a representative of a gold nanoparticle carrier and positioned in front of the various heteroatoms present in etravirine. After that, the electronic energy and zero-point energy of both the gold atom and etravirine were calculated. Next, both entities were brought into proximity at an interaction distance of 2.8 angstroms. The energy of the resulting complex was determined and penalized by the sum of the energies of the individual monomers. The results, presented in Fig 8, indicate that the lowest energy (-6.6 kcal/mol) complex was observed when the gold atom was placed in front of nitrogen number 8. Generally, the placement of the gold atom at different sites around the heteroatoms yielded stable complexes with low energy, suggesting their potential as guides for experimental gold nanoparticle carriers for etravirine. The interaction between the gold atom and etravirine’s heteroatoms involved non-covalent bonding. This preliminary non-covalent interaction could facilitate the release of the drug from the designed nanoparticle carrier upon reaching the active site in *in vivo* settings [66]. Gold-based nanoparticle delivery systems are well-known for their ability to alleviate clinical manifestations of cancer while reducing the side effects associated with chemotherapy administration [67,68].

Reported clinical assessments have concluded that etravirine exhibits an acceptable safety profile [69]. Combined with the results obtained from molecular docking, MM/GBSA, and MD, these findings suggest that etravirine holds promise as a potential candidate against TNBC. Furthermore, the DFT calculations between etravirine and the gold atom can serve as a foundation for the design of a targeted drug delivery system, facilitating the accumulation of the candidate drug at the tumour site in effective inhibitory concentrations while minimizing adverse effects.

## Conclusion

The primary objective of this research study was to identify approved drugs that can effectively combat TNBC using computational techniques such as molecular docking, MD, and DFT calculations. Through molecular docking and MM/GBSA analysis, three drugs (sunitinib, bazedoxifene, and etravirine) were identified as having a higher binding affinity towards the ck2 alpha protein compared to the co-crystallized inhibitor. Further refinement of the three drugs revealed that only etravirine, an antiviral medication, exhibited a more stable and reliable binding mode with the protein than the reference ligand (inhibitor 108600). Etravirine is known to have a safe clinical profile. DFT quantum mechanical calculations were conducted to determine the interaction energy between etravirine and a representative gold atom, resulting in a stable interaction energy. This finding suggests a potential formulation of the drug as a gold nanoparticle for intravenous delivery in TNBC patients. The *in silico* results provided in this study indicate that etravirine could be repurposed in TNBC treatment regimens after further laboratory and consequent clinical assessments and trials.

## Supporting information

S1 Data (XLSX)

S1 File (DOCX)
