# Supplementary material for: Exploring the potential of approved drugs for triple-negative breast cancer treatment by targeting casein kinase 2: Insights from computational studies
Source: PLoS One. 2023 Aug 14;18(8):e0289887. doi: 10.1371/journal.pone.0289887 (PMC10424868; doi:10.1371/journal.pone.0289887)
Supplement: S1 File — (DOCX) [file pone.0289887.s002.docx]

| Molecule | Docking score  Kcal/mol | MMGBSA dg binding energy | Rotatable bonds |
| --- | --- | --- | --- |
| Sunitinib | -10.401 | -62.16 | 8 |
| Bazedoxifene | -7.937 | -33.56 | 9 |
| Etravirine | -7.743 | -48.38 | 4 |
| Idelalisib | -6.931 | -33.33 | 5 |
| Pitavastatin | -6.274 | -34.67 | 10 |
| Vismodegib | -5.638 | -39.19 | 5 |
| Dimethyl Fumarate | -1.340 | -34.83 | 4 |
| Refercence ligand | -7.390 | -51.209 | 5 |

**Table** summarizing the docking scores and MMGBSA binding energies of the studied approved drugs
